# Sleep Disorders, Obesity, Hypertension, and Cardiovascular Risk

**DOI:** 10.1155/2015/197534

**Published:** 2015-10-18

**Authors:** Samy I. McFarlane, Olugbenga Ogedegbe, Amgad N. Makaryus, Charles Agyemang, Girardin Jean-Louis

**Affiliations:** ^1^Downstate Medical Center and Kings County Hospital Center, State University of New York, Brooklyn, NY, USA; ^2^Langone Medical Center, New York University, NY, USA; ^3^Department of Cardiology, North Shore-LIJ Health System, Hofstra North Shore-LIJ School of Medicine, Nassau University Medical Center, East Meadow, NY 11554, USA; ^4^Department of Public Health, Academic Medical Centre, University of Amsterdam, 1105 AZ Amsterdam, Netherlands

The pandemic of obesity is associated with parallel epidemics of sleep disorders, diabetes, hypertension, and cardiovascular disease (CVD), that is, the primary cause of morbidity and mortality among adults [1]. Sleep disorders generally go unrecognized and undiagnosed and appear to play a major role in the interrelationships between obesity, diabetes, hypertension, and cardiovascular disease [2–4]. Accumulating evidence from cross-sectional as well as longitudinal studies by various groups, including ours, indicates close interrelationships among sleep disorders and diabetes, hypertension, and CVD including stroke, coronary artery disease, and heart failure, particularly among minority populations and women [2–5]. Postulated mechanisms or mediators of interrelationships between sleep disorders and CVD include oxidative stress, increased inflammation, increased uric acid endothelial dysfunction, dyslipidemia, and hypercoagulability, which are common underlying CVD risk factors among individuals with metabolic syndrome [6–8]. Furthermore, the cost of undiagnosed sleep disorders appears to be exceedingly high, prompting the American Academy of Sleep Medicine to recommend screening for at-risk individuals [9, 10]. This is quite important giving the mounting evidence of decreased CVD risk and improved cardiometabolic functions with treatment of sleep disorders, particularly among patients with sleep apnea [9].

In this special issue, we assembled a group of world-renowned editors with complementary expertise in sleep medicine, hypertension, diabetes, obesity, and cardiovascular disease to lead this initiative. Commensurate with level of expertise of the editorial team, we were able to attract important papers from established investigators from all over the world. Judging from the accepted papers for this special issue, we surmise that they are both topical and timely and they are likely to have a significant impact on the field.

The issue covers a wide range of topics, from dietary interventions, in the form of low glycemic index food and its effectiveness in lowering blood pressure to factors associated with medication nonadherence among hypertensives in two African countries: Ghana and Nigeria. Another important topic covered is the implications of renal denervation, a rather novel and increasingly studied potential therapy for hypertension, in patients with sleep apnea. An interesting topic also examined in a research paper indicated that in Black American working nonday time shift are more likely to report hypertension, especially with short sleep duration. This article opens further questions for investigations including those examining the underlying mechanisms for these findings. This special issue also included an epidemiologic assessment for the prehypertension and hypertension in two countries in Sub-Saharan Africa. This study assessed predictors of various stages of hypertension including obesity and educational levels in these vulnerable populations. Another closely related article from Africa among people with hypertension assessed the subtypes of hypertension in different light including controlled hypertension, isolated systolic hypertension, and isolated diastolic hypertension as well as systolic-diastolic hypertension as they relate to obesity. This is quite important since different subtypes confer various degrees of risk. For example, among Blacks and/or people with diabetes systolic hypertension is predominant and is associated with high CVD risk factors including microalbuminuria, insulin resistance, postural hypertension, salt sensitivity, and volume expansion. Characterization of subtypes of hypertension not only opens the doors for further investigations of research questions, but also helps strategize in terms of treatment such as the utilization of low salt diet, diuretics, and agents that inhibit the renin angiotensin aldosterone function (RAAS), such as Angiotensin Converting Enzyme (ACE) Inhibitors and the Angiotensin Receptor Blockers (ARBs).

Finally, the interrelationships between sleep disorders and CVD are explained and illustrated in a well-written review article included in this issue that highlights the most recent findings and insights in this highly complex topic, providing the readers with food for thought that will hopefully generate testable and clinically important hypotheses.

*Samy I. McFarlane* *Samy I. McFarlane* *Olugbenga Ogedegbe* *Olugbenga Ogedegbe* *Amgad N. Makaryus* *Amgad N. Makaryus* *Charles Agyemang* *Charles Agyemang* *Girardin Jean-Louis* *Girardin Jean-Louis*
